# Research on the wound healing effect of Shengji Huayu Formula ethanol extract-derived fractions in streptozotocin-induced diabetic ulcer rats

**DOI:** 10.1186/s12906-023-03894-0

**Published:** 2023-03-01

**Authors:** Jing-Ting Zhang, Min-Feng Wu, Ming-Hua Ma, Liang Zhao, Jian-Yong Zhu, Hua Nian, Fu-Lun Li

**Affiliations:** 1 Department of Dermatology, Yueyang Hospital of Integrated Traditional Chinese and Western Medicine, Shanghai University of Traditional Chinese Medicine, Shanghai, 200437 China; 2 Department of Pharmacy, Yueyang Hospital of Integrated Traditional Chinese and Western Medicine, Shanghai University of Traditional Chinese Medicine, Yueyang, 200437 China; 3 Department of Ophthalmology, Shanghai General Hospital (Shanghai First People’s Hospital), Shanghai Jiao Tong University, Shanghai, 200080 China; 4 Department of Dermatology, Huadong Hospital, Fudan University, Shanghai, 200040 China; 5 Department of Pharmacy, Yangpu Hospital, Tongji University School of Medicine, Shanghai, 200090 China; 6 Department of Pharmacy, Shanghai Baoshan Luodian Hospital, Shanghai, 201908 China; 7 Department of Pharmacy Research, Yueyang Hospital of Integrated Traditional Chinese and Western Medicine, Shanghai University of Traditional Chinese Medicine, Shanghai, 200437 China

**Keywords:** Shengji huayu formula, Diabetic ulcer, Chloroform extract, Chemical constituents

## Abstract

**Background:**

Diabetic ulcer is a common complication of diabetes. It is characterized by a long-term disease course and high recurrence rate. Shengji Huayu Formula (SHF) is an effective formula for treating diabetic ulcers. However, the specific effective parts of SHF remain unclear. Clarifying the active polar site of SHF would be helpful to refine research on the components in SHF that promote wound healing. This research aims to focus on evaluating the activity of polar fractions.

**Methods:**

A diabetic rat model was established by intraperitoneally injecting streptozotocin (STZ) and was adopted to confirm the therapeutic effect of SHF. Four different polarity parts were extracted from SHF and prepared into a cream to evaluate the activity. High-performance liquid chromatography (HPLC) was used to detect chemical constituents in chloroform extracts.

**Results:**

It was discovered that dracorhodin, aloe-emodin, rhein, imperatorin, emodin, isoimperatorin, chrysophanol, physcion, and tanshinone IIA were the main components of the chloroform extract from SHF. The results revealed that chloroform extract could effectively accelerate diabetic wound healing by promoting collagen regeneration and epidermal repair. Chloroform extract of SHF could stimulate the generation of vascular endothelial growth factor (VEGF). The results are also indicated that the effective active fraction was the chloroform part, and the method of detecting the main chemical constituents in the active part was successfully established.

**Conclusion:**

SHF could improve diabetic ulcers by promoting granulation tissue synthesis. In this study, four polar parts (petroleum ether, chloroform, ethylacetate, n-butanol) were extracted from a 95% ethanol extract. In contrast, chloroform polar parts showed a higher wound closure rate, stimulated more collagen regeneration and promoted more production of vascular endothelial cells. In conclusion, the chloroform extract of SHF was the effective polar part in ameliorating diabetic wound healing.

## Background

Diabetes mellitus (DM) is a type of endocrine disease that is estimated to affect 284.6 million people worldwide [1]. The International Diabetes Federation (IDF) reported that 451 million people suffered from diabetes in 2017, and this number is expected to increase to 693 million by 2045 [2]. Diabetic ulcer, one of the most common complications of DM, is a kind of chronic and refractory cutaneous ulcer with several characteristics, including long-term refractoriness, easy recurrence and high incidence. In China, the morbidity of diabetic ulcers has reached 8.1% [3]. It is estimated that chronic trauma causes losses of over $25 billion a year in the U.S., leading to an increase in medical costs [4, 5]. Diabetic ulcers cause great pain and a heavy economic burden to patients. Therefore, studying effective drugs and treatment methods to improve diabetic ulcers is an urgent task. At present, anti-infection, hyperbaric oxygen therapy and surgery are common treatments in the clinic. However, these therapies have several limitations, including a slow effect and difficulties in scab formation. Traditional Chinese medicine (TCM) has advantages in treating diabetic ulcers [6, 7]. External treatment has been a characteristic TCM therapy since ancient times. The effective substances and chemical constituents of TCM play a vital role in the healing of diabetic ulcers. Shengji Huayu Formula (SHF) has been applied in the clinic for decades and is a safe and effective therapy for treating chronic cutaneous ulcers, especially diabetic wounds. SHF could reduce wound closure time with subtle pain and low treatment expenditure [8–11]. SHF can improve local blood circulation and accelerate wound granulation, epithelium regeneration and healing processes [8–11]. However, the effect of the active polar component of this formula remains unknown.

We previously used several animal models to assess the active effects of SHF, including the high-fat diet-induced diabetic ulcer model to assess the 95% ethanolic extract [11], female clean-grade diabetic mouse models to evaluate the 70% ethanolic extract [12], and STZ-induced animal models to assess the 95% ethanolic extract [13]. All animal experiment results showed that SHF ethanol extract could accelerate re-epithelialization and reduce diabetic mouse wound healing time. In in vitro studies, human dermal microvascular endothelial cells (HDMECs) and shRNA interference were used to explore the effects of SHF ethanol extract on cell migration, PGT, PGE2, and the angiogenesis factor VEGF. Our in vitro studies confirmed that SHF ethanol extract could accelerate re-epithelialization and reduce inflammation by regulating the Activin/Follistatin imbalance [11, 12]. In addition, the molecular mechanisms of SHF in treating diabetic ulcers were revealed by transcriptional profiling and network analysis in recent years [9, 10]. All of these studies explain the SHF mechanism from numerous perspectives, but the active polar parts of the SHF ethanol extract need to be investigated further.

Thus, this research was designed to verify the active polar parts in SHF with the function of promoting wound healing. The most active part, the chloroform fraction, was established as the HPLC method to detect the main chemical constituents. Comprehensive and objective evaluation of the chemical constituents in SHF contributes to providing scientific evidence for diabetic ulcer treatment.

## Materials and methods

### External TCM ointment preparation

**Fig. 1:**
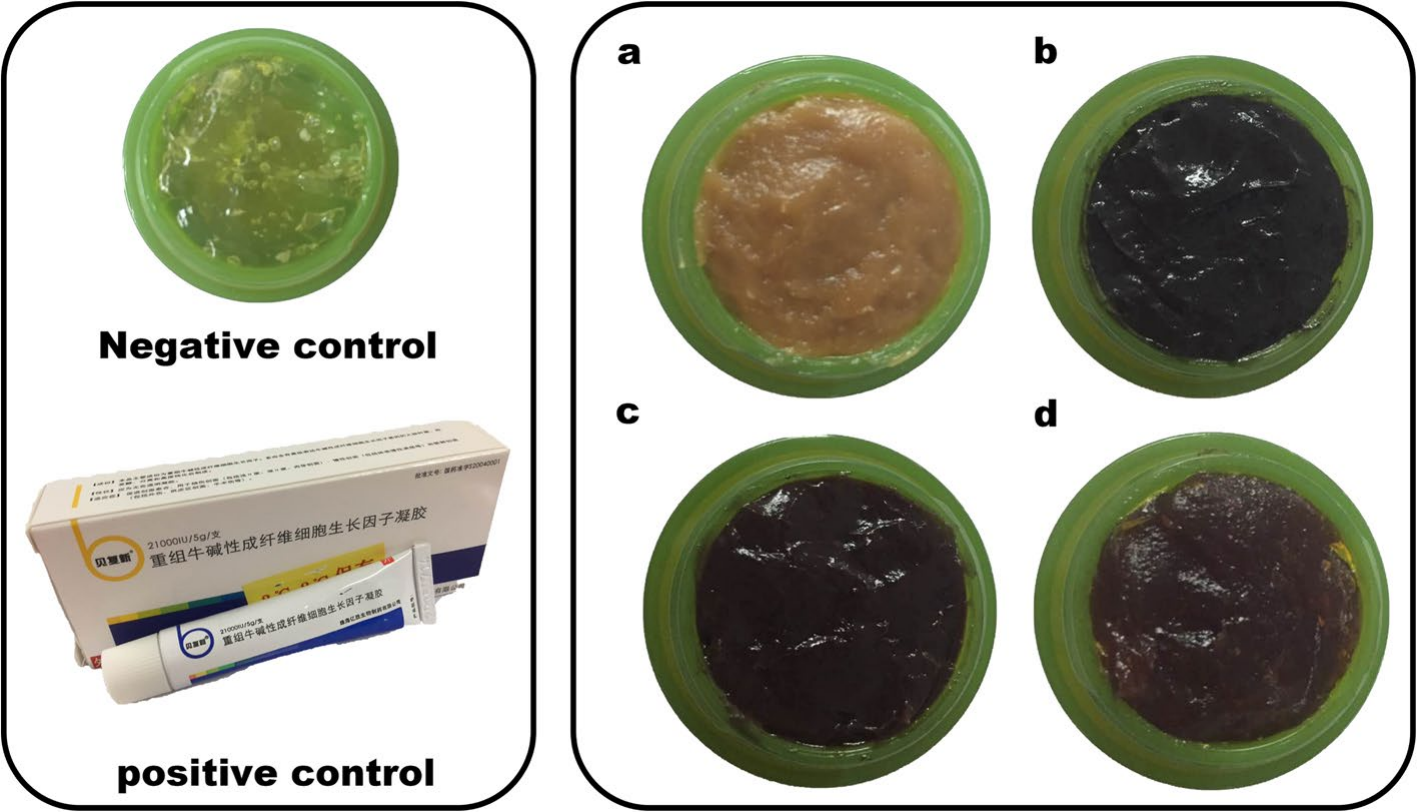
Experimental drugs used in negative control group, positive control drug group and four polar parts group. Take carbomer as substrate. Left (up): negative control (carbomer) and positive control (rb-bFGF). Right: four polarity components **a** Petroleum ether-extract; **b** Chloroform-extract; **c** Ethyl acetate-extract; **d** *n*-butanol-extract

SHF contained eight Chinese herbs, as shown in Table 1. The dosage used in the present study was determined according to the *Chinese Pharmacopoeia* (2015 edition). The air-dried powder of herbs in SHF was extracted three times with 95% ethanol at room temperature to produce a crude extract upon removal of the solvent. The extract was suspended in water and partitioned successively with petroleum ether, chloroform, ethyl acetate, and *n-*butanol at a 1:1 ratio 4 times to afford four corresponding portions. The four concentrated solutions were evaporated in vacuo to produce a semisolid residue, which was mixed with swollen carbomer and triethanolamine to adjust the pH value to 6–8, labeled and stored in a refrigerator at 4 °C. Therefore, four polar fractions were prepared as shown (Fig. 1). As a negative control, the blank gel matrix, 0.60 g carbomer, and 0.50 g glycerin were dissolved in 18 mL water and were swelled overnight. The positive control was recombinant bovine basic fibroblast growth factor (rb-bFGF).

**Table 1:** The composition of SHF

Latin scientific name	Chinese name	Plant part	Weight (g)	%
*Astragalus membranceus* (Fisch.) Bge	Huangqi	Radix	30.0	15.8
*Salvia miltiorrhiza* Bge	Danshen	Rhizoma	15.0	7.9
*Angelica dahurica* (Fisch ex Hoffm.) Benth. et Hook. f	Baizhi	Radix	30.0	15.8
*Rheum palmatum* L	Dahuang	Rhizoma	15.0	7.9
*Daemonorops draco* Bl	Xuejie	Resin	10.0	5.3
*Arnebia euchroma* (Royle) Johnst	Zicao	Radix	30.0	15.8
*Pteria martensii* (Dunker)	Zhenzhufen	/	30.0	15.8
*Calamine*	Luganshi	/	30.0	15.8

### Animal

Sprague Dawley (SD) rats (8 weeks old, 150 ± 5 g) were obtained from Shanghai SLAC Laboratory Animal Co., Ltd. (SLAC Shanghai 2012–0002) and kept under standard temperature (25 °C) in the laboratory of Shanghai University of Traditional Chinese Medicine. In total, 96 male SD rats were randomly divided into six groups (excluding rats that died in the process of modeling and whose blood glucose did not meet the standard), 16 in the negative control group, 16 in the positive control (rb-bFGF), 10 in the petroleum ether group, 14 in the chloroform group, 10 in the ethyl acetate group, and 14 in the *n*-butanol group. In this study, rats were anesthetized with isoflurane and a small animal anesthesia machine. After the experiments were finished, all rats were placed in a closable box and euthanized by CO_2_ inhalation to be suffocated. After CO_2_ inhalation, rats were subjected to cervical dislocation with eyeball whitening, heart failure and respiratory arrest, which confirmed death. The study on rats was approved by the ethical committee of Shanghai University of Traditional Chinese Medicine (No. 16661, 16,702).

### Diabetic wound model

Diabetic rat models were established according to the classic modeling method [14]. After 3 days of acclimatization, the 8‑week‑old mice were fed a high-fat and high-sugar diet consisting of 54.6% basic mouse feed, 16.9% lard, 14% sugar, 10.2% casein, 2.1% premix, and 2.2% maltodextrin for two weeks. Then, the diabetic model was induced by intraperitoneal injection of 1% STZ solution of 50 mg/kg and intragastric administration of 10% glucose solution 2 h later to balance blood glucose. The weight of the rats was measured on the day of modeling, and tail tip blood was collected to measure blood glucose.

One week after the last STZ injection, rats with blood glucose levels over 16.7 mmol/L, polyuria, polydipsia and intense hunger symptoms were considered to have successfully developed diabetes mellitus. On the day of modeling, rats were weighed and anesthetized with 2%-3% isoflurane. After anesthesia, the model area (both sides of the spine) was depilated with a depilatory knife. Under aseptic conditions, skin wounds with a diameter of 0.6 cm were made into diabetic rats with an area of 0.28 cm^2^. Each rat had 6 holes on the back. The depth of the wound reached the level of the subfascial dressing, and every rat was fed continuously in an individual cage separately. Intergroup markers should be made for high-fat and high-sugar diets.

### Drug delivery and specimen collection

The rats in the petroleum ether group, chloroform group, ethyl acetate group, *n*-butanol group, positive control group, and negative control group were treated by intergroup comparison. After the model was established, the rats in the treatment group were treated with four polar parts of SHF, while the rats in the positive control group were treated with rb-bFGF, and the rats in the negative control group were treated with carbomer. The ointment was applied at a dose of 0.5 g/cm^2^/day immediately after the punch, and wound dressing was performed once a day. The wound was left uncovered. On Days 3, 7, and 9 after model establishment, the wound area of the rats was measured. On Day 9, half of the rats in each group were euthanized by CO_2_ inhalation, and the remaining rats were sacrificed after healing. A piece of basal muscle tissue of the wound was cut quickly. Two wounds in each group were fixed with 4% paraformaldehyde, and 75% alcohol was exchanged after 24 h. The remaining wounds were placed in the refrigerator at − 80 ℃.

### Wound healing process evaluation

The wound area of the rats was recorded on Days 3, 7, 9, and 11. The measurement of wound area was conducted by visual inspection, digital camera and ImageJ software. First, a circular piece of paper with a diameter as a standard reference was placed over the wound. A digital camera was used to record the wound shape and outline from a fixed distance. Then, ImageJ 1.49v was adopted to analyze and evaluate the size of the wound according to wound photos at Day 9. ImageJ can calculate the irregular wound area by extracting the background color, enhancing the color difference and accumulating the sum of pixels. The area data were obtained, and the percentage of wound area was calculated as W_C_ %.

Wound closure (%) = (1 − W_C_)/W_O_ × 100%.

W_C_: wound area at the current observation time point.

W_O_: original wound area.

### Histological examination by HE staining

The collected samples were dehydrated with different concentrations of ethanol, embedded and sliced at a thickness of 5 µm. The samples were baked for 1 h in a 60 ℃ thermostat, paraffin was removed three times with xylene, 10 min each time, washed twice, 5 min each time, and treated with Harris hematoxylin staining for 5 min. Then, the samples were washed with water for 5 min, differentiated by 1% hydrochloric acid alcohol solution for 5 s, washed with tap water for 15 min and stained with 0.5% eosin (water solubility) for 1 min, 80% ethanol for 2 min, 95% ethanol 2 times for 5 min each time, and 100% ethanol 2 times for 5 min each time. Finally, xylene was transparently treated twice, 5 min each time, 1–2 drops of gum were added, and glasses were added to seal it. After the samples were collected, they were placed into 4% formalin solution. The samples were then paraffin-embedded, sectioned, and stained with hematoxylin and eosin (HE). Histopathological changes were observed under a light microscope.

### Immunohistochemical staining and evaluation

The wounds were resected immediately after the rats were killed and fixed in 4% neutral buffered paraformaldehyde at 4 °C for 24 h. Selected samples were embedded in paraffin, sectioned 5 µm thick, deparaffinized, and rehydrated with PBS (pH 7.4), and the antigen was retrieved with high temperature and pressure for 5 min, incubated with aqueous 3% H_2_O_2_-methanol for 10 min, washed with PBS 3 times × 5 min, and stained serially with anti-PCNA and anti-VEGF at 4 °C overnight. The slices were incubated with a secondary antibody of IgG-HRP at 37 °C for 60 min, washed with PBS 3 times × 5 min, and incubated with DAB for 5 min. The reaction was terminated with water for 15 min and counterstained with hematoxylin. Sections were mounted with 1–2 drops of gum after transparency with xylene. For the nuclear staining protein PCNA and cytoplasmic staining protein VEGF, semiquantitative analysis was conducted using ImageJ software. Percentage-positive cells were calculated as the number of positively stained cells × 100/total number of cells in photomicrographs of tissue. The percentage of positive cells was calculated in a high-power field (HPF) (magnification 400 ×) and repeated for 10 HPFs. The arithmetic mean ± standard error deviation of counts was used for statistical analysis.

### Preparation of standard and sample solutions of chloroform for HPLC

To certify the HPLC method, a standard stock solution was prepared and treated with a gradient mixed reference solution in methanol to the spiked concentration (2.58–258 µg/mL for dracorhodin perchlorate, 1.86–185.71 µg/mL for aloe emodin, 2.26–226 µg/mL for rhein, 1.31–131 µg/mL for imperatorin, 3.06–306.43 µg/mL for emodin, 4.42–442 µg/mL for isoimperatorin, 4.14–414 µg/mL for chrysophanol, 1.59–159.29 µg/mL for physcion, 1.63–162.86 µg/mL for tanshinone IIA). For the determination of the chloroform part of the SHF, the concentrated solution was extracted with chloroform at a 1:1 ratio 4 times, and the extractions were mixed together, concentrated to 0.72 g/mL by rotary evaporation, and then diluted to a proper concentration. Three replicates were used for each sample. All standards and sample preparations were filtered through a 0.45 µm membrane filter before injection into the HPLC system for analysis.

### Equipment and chromatographic conditions

Chromatographic column: Agilent 1100 series HPLC, Agilent Zorbax Eclipse XDB-C18 (4.6 mm × 250 mm, 5 µm), column number: 990967–902, mobile phase: A phase is acetonitrile, B phase is 0.2% formic acid aqueous solution (0 min-10 min: 30% A, 50 min: 45% A, 60 min: 50% A, 75–90 min: 65% A, 95–110 min: 95% A), flow rate: 1 mL/min. The detection wavelength was 254 nm. The column temperature was 30 ℃. The injection volume was 10 µL, and the gradient of mobile phase was used as the initial condition to balance 20 min before injection.

### Statistical analysis

SPSS 21.0 software was used for statistics, and data are expressed as the mean ± SEM. Statistical analysis for differences among groups was tested by one-way ANOVA with Dunnett’s or Tukey’s multiple comparisons test. Statistically significant results were expressed as *p* < 0.05.

## Results

### General observation and random blood glucose

**Fig. 2:**
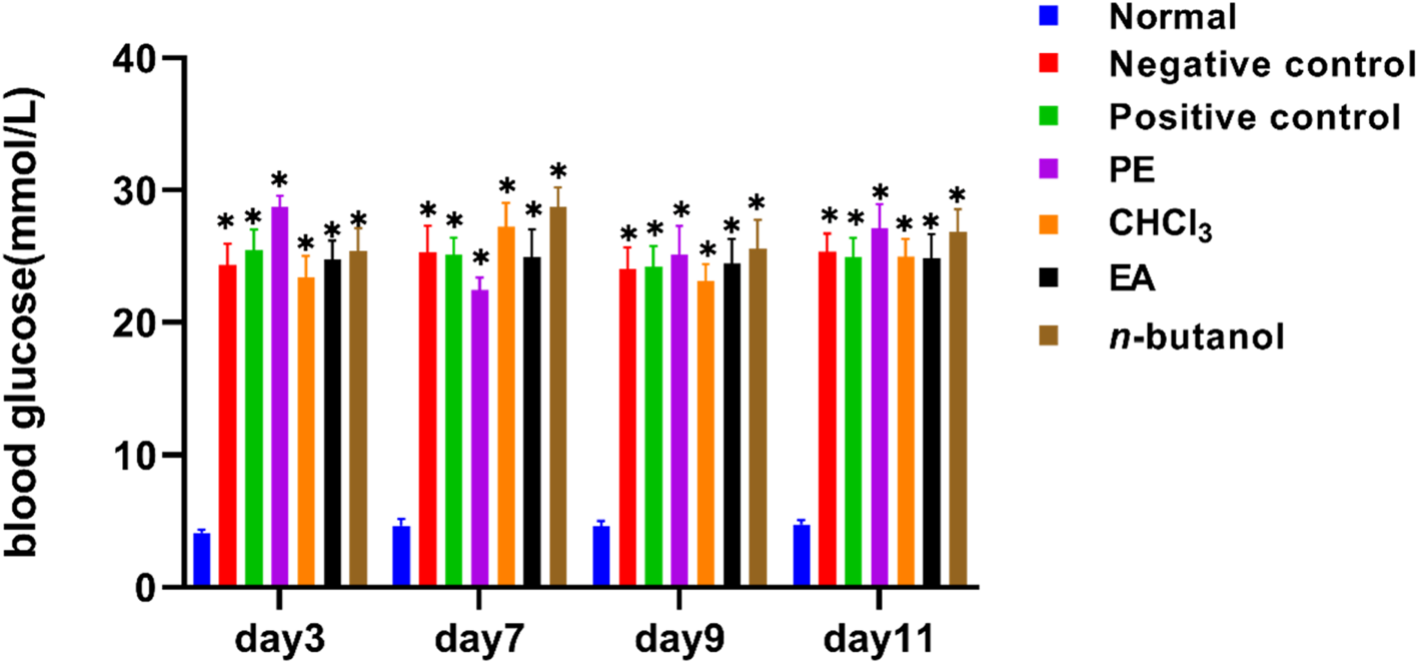
The blood glucose values of rat tail vein were stable after 3, 7, 9, and 11 days of wound modeling. Normal: Before modeling; Negative control:carbomer; Positive control:rb-bFGF; PE: Petroleum ether-extract; CHCl_3_: Chloroform-extract; EA: Ethyl acetate- extract; *n*-butanol: *n*-butanol-extract. ^*^p < 0.05 vs normal

Body weight was reduced, and intake, water intake and excretion were significantly increased, which was in accordance with the classic characteristics of diabetes mellitus. The random blood glucose of the tail vein of rats was stable after 3, 7, 9, and 11 days of wound modeling (Fig. 2).

### Analysis of the wound closure area and the active polar parts of the SHF

**Fig. 3:**
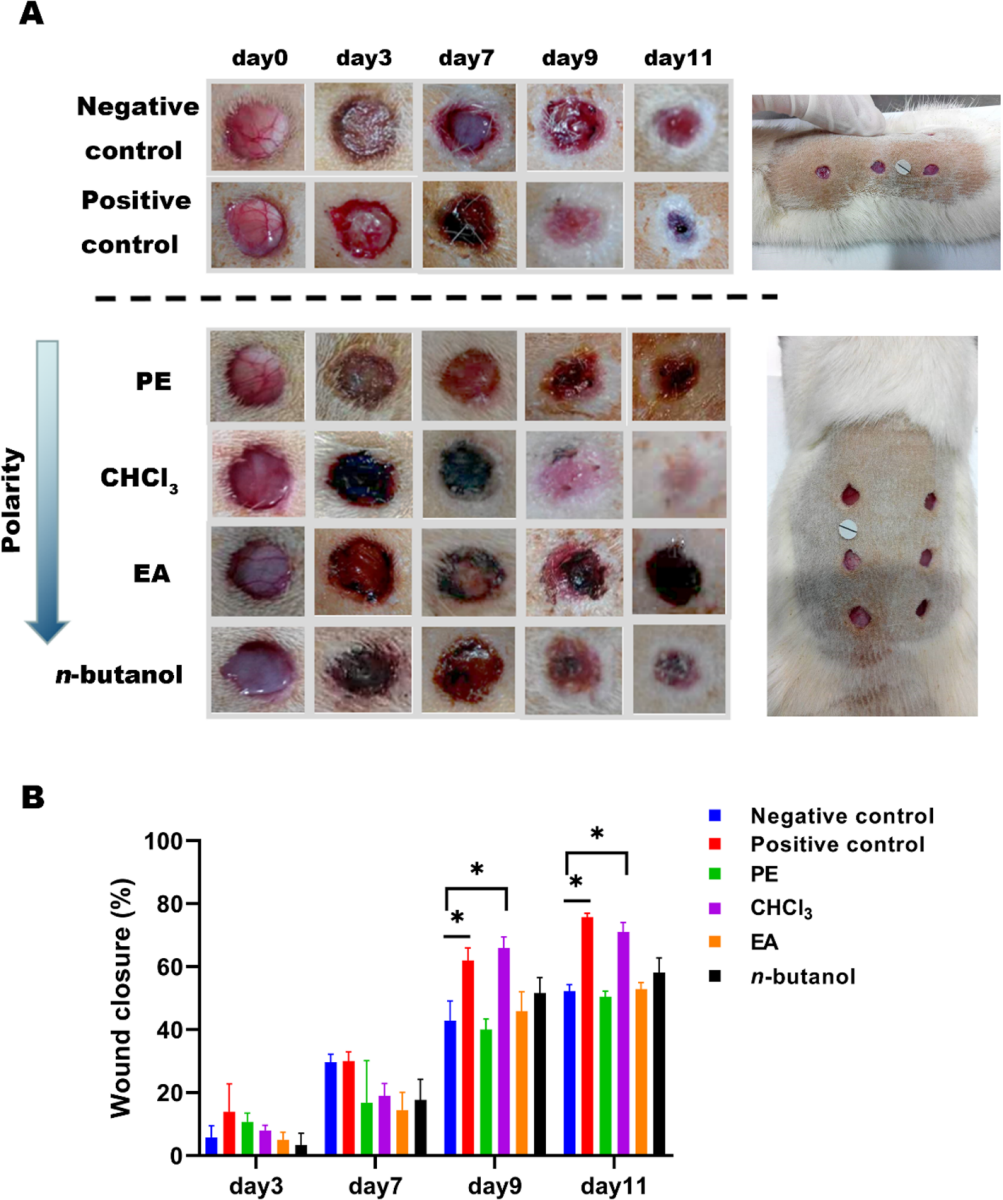
Photographic representation of wound closure on different post wounding days. **A** The morphological changes of polarity components wound at different time points. Wounds were marked with a ruler and photographed by a camera on day 3, 7, 9, and 11 after rat were modeled. **B** Effect of polarity components wound area ratio of each group at day 3, 7, 9, and 11 (Mean ± SEM). Negative control:carbomer; Positive control:rb-bFGF; PE: Petroleum ether-extract; CHCl_3_: Chloroform-extract; EA: Ethyl acetate-extract; *n*-butanol: *n*-butanol-extract. ^*^p < 0.05 vs negative control group

On Days 3 and 7 of administration, no significant difference was observed in the wound area (Fig. 3) between the four polar parts (petroleum ether, chloroform, ethylacetate, and *n*-butanol) group and the negative control group (*p* > 0.05) or among the four polar parts (*p* > 0.05). On Days 9 and 11, the healing degree of wounds in the positive control group and chloroform group was significantly different from that in the negative control group (*p* < 0.05). Significant differences were discovered between the chloroform group and four other groups (negative control, petroleum ether, ethyl acetate, and *n*-butanol groups) (*p* < 0.05).

### HE staining of active polar parts of SHF

**Fig. 4:**
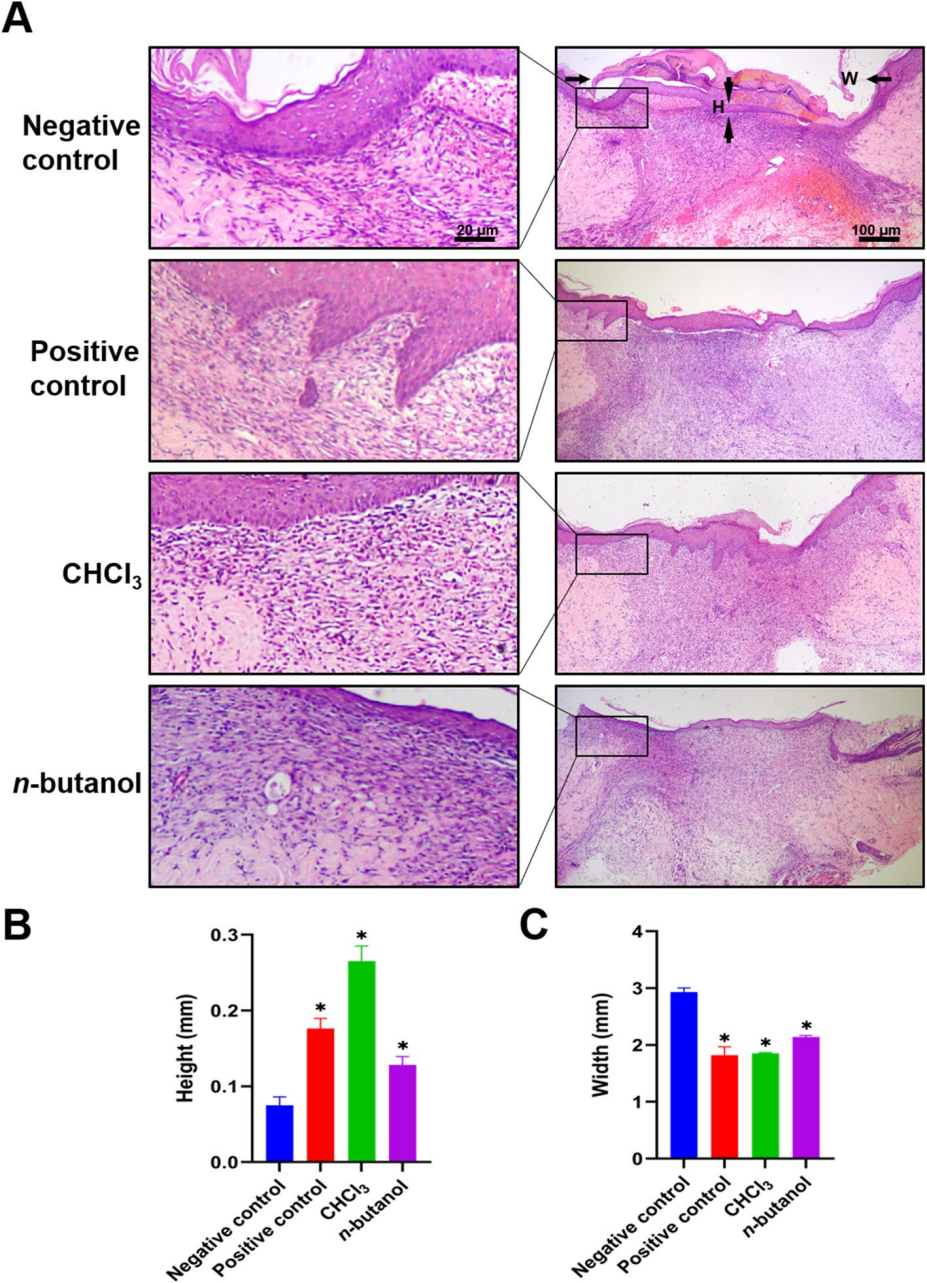
**A** Effect of HE staining of wound granulation tissue at day 9. **B** and **C** The epidermis thickness and healing width at day 9. Negative control:carbomer; Positive control:rb-bFGF; CHCl3: Chloroform-extract; n-butanol: n-butanol-extract. *p < 0.05 vs negative control

The results of HE staining on Day 9 showed that the wound epidermis of the negative control group was thin, and collagen in the dermis was loose with blurred cell layers and irregular layers. In contrast, the epidermis of the positive control group was obviously much thicker, and the repair condition was more complete with no necrotic tissues. In addition, the wound epidermis of the chloroform group had the best repair condition with the thickest epidermis layer and most abundant collagen regeneration. However, collagen tissues of the *n*-butanol group were incomplete with little collagen regeneration and epidermis layer (Fig. 4A). As a result, there was a significant difference in wound healing width and epidermal thickness between the four groups (*p* < 0.05, Fig. 4B and 4C).

### PCNA staining of active polar parts of SHF

**Fig. 5:**
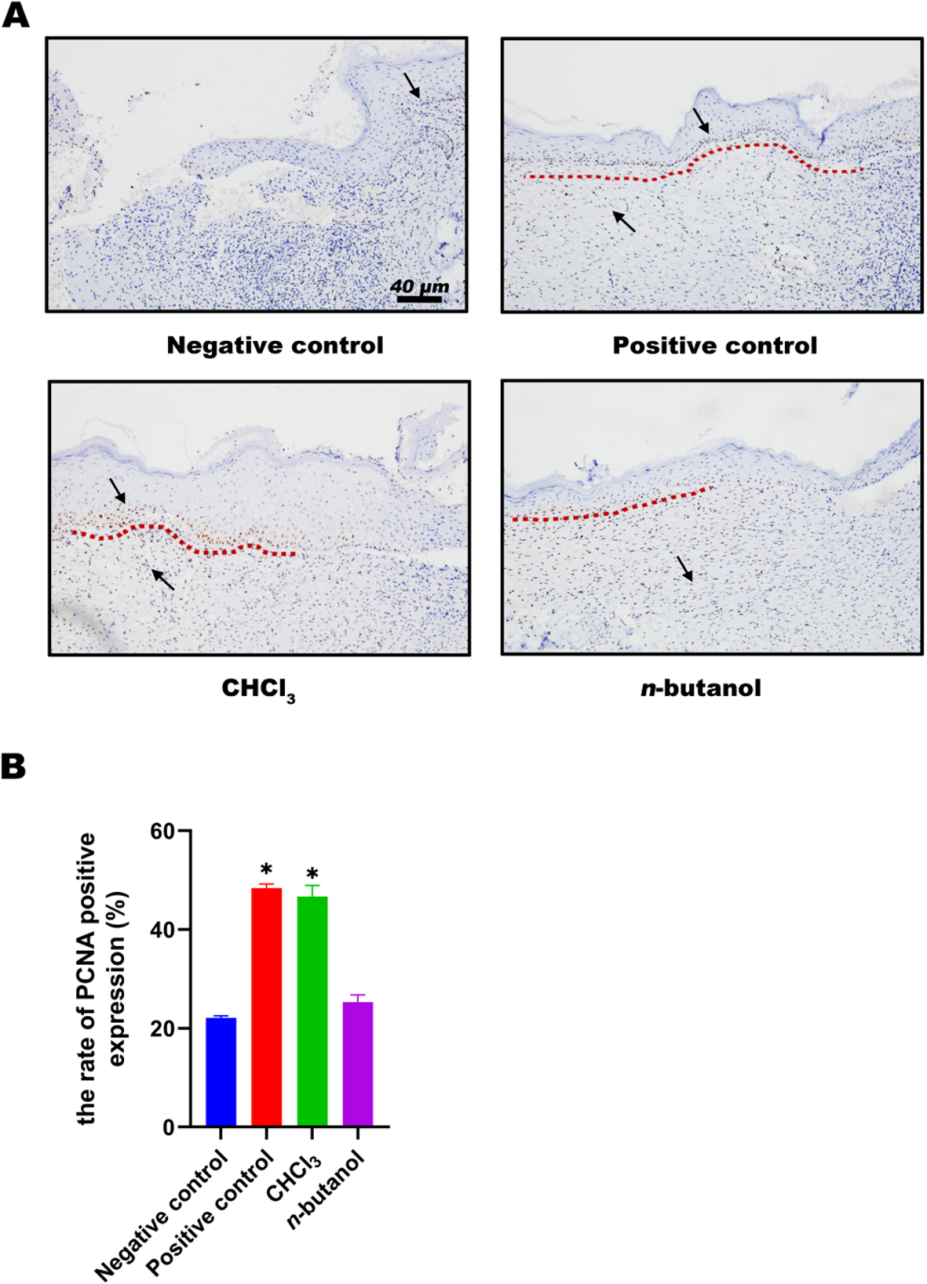
**A** Effect of PCNA staining of wound tissue at day 9. Red dotted lines describe cell proliferation and positive cells marked in black arrows in the pictures. **B** PCNA positive expression of wound tissue at day 9. Negative control:carbomer; Positive control:rb-bFGF; CHCl3: Chloroform-extract; n-butanol: n-butanol-extract. *p < 0.05 vs negative control

On Day 9, proliferating cell nuclear antigen (PCNA) staining results showed that cell layers in the negative control group were blurred and irregular with few PCNA-positive cells. In the positive control group, dermal cells repaired well with regularly arranged positive cells. In the chloroform group, the best condition for epidermal repair was observed, and the epidermal layer was obviously much thicker. Positive cells were regularly arranged, and collagen regeneration was obvious. In the *n*-butanol group, collagen tissues exhibited a poor repair condition of few positive cells, and the arrangement was comparatively regular (Fig. 5A). As a result, the chloroform group and positive drug group contained significantly more PCNA-positive cells than that of the negative control group (*p* < 0.05, Fig. 5B).

### VEGF staining of active polar parts of SHF

**Fig. 6:**
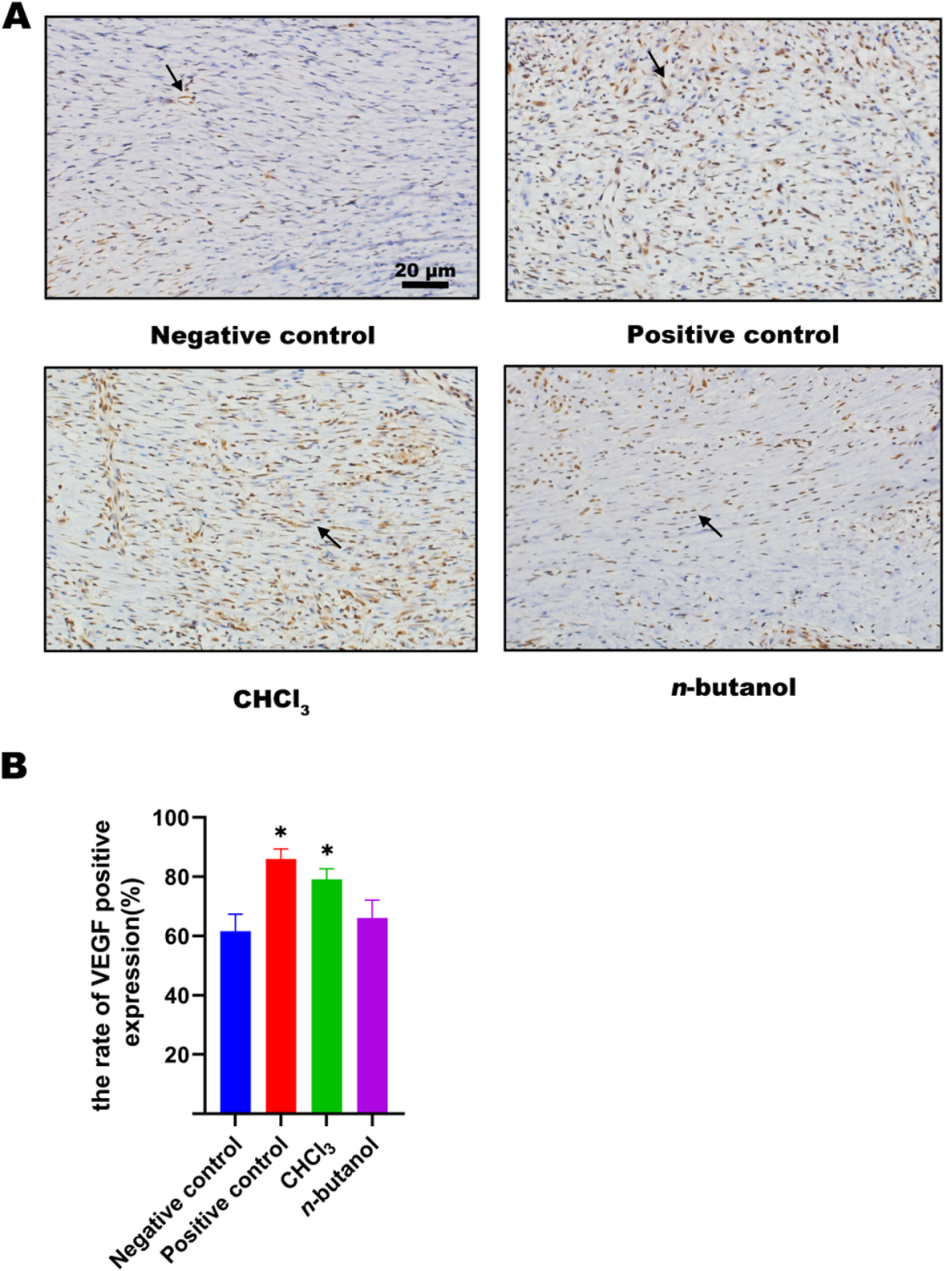
**A** Effect of VEGF immunohistochemistry at day 9. Positive cells marked in black arrows in the pictures. **B** VEGF positive expression of wound tissues at day 9. Negative control:carbomer; Positive control:rb-bFGF; CHCl3: Chloroform-extract; n-butanol: n-butanol-extract. *p < 0.05 vs negative control

On Day 9, the staining results showed that the vascular endothelial growth factor (VEGF)-positive cells became brown. The negative control group showed little positive expression of VEGF with irregular stratification and few vascular endothelial cells. In the positive control group, positive expression of VEGF was observed with abundant vascular endothelial cells. In the chloroform group, VEGF-positive cells were accompanied by regularly arranged vascular endothelial cells, complete repair of endothelial cells and much collagen regeneration. In the *n*-butanol group, the collagen tissue was comparatively repaired, and compared to the chloroform group, VEGF expression and positive cell arrangement were poorer (Fig. 6A). As a result, the number of VEGF-positive cells indicated the number of vascular endothelial cells within different groups. There were significantly more vascular endothelial cells in the chloroform group and the positive drug group than in the negative control group (*p* < 0.05) (Fig. 6B).

### HPLC analysis of the chloroform extract of SHF

Dracorhodin is the main compound in *Daemonorops draco* Bl., and the main compounds in *Rheum palmatum* L. are aloe-emodin, rhein, emodin, chrysophanol, and physcion. Imperatorin and isoimperatorin are the main compounds in *Angelica dahurica* (Fisch ex Hoffm.) Benth. et Hook. f., and tanshinone IIA is the main compound in *Salvia miltiorrhiza* Bge. The nine compounds are also used by the Chinese Pharmacopoeia to assess the quality of *Daemonorops draco* Bl., *Rheum palmatum* L., *Angelica dahurica* (Fisch ex Hoffm.) Benth. et Hook. f., and *Salvia miltiorrhiza* Bge., respectively (Chinese Pharmacopoeia Commission 2015).

**Fig. 7:**
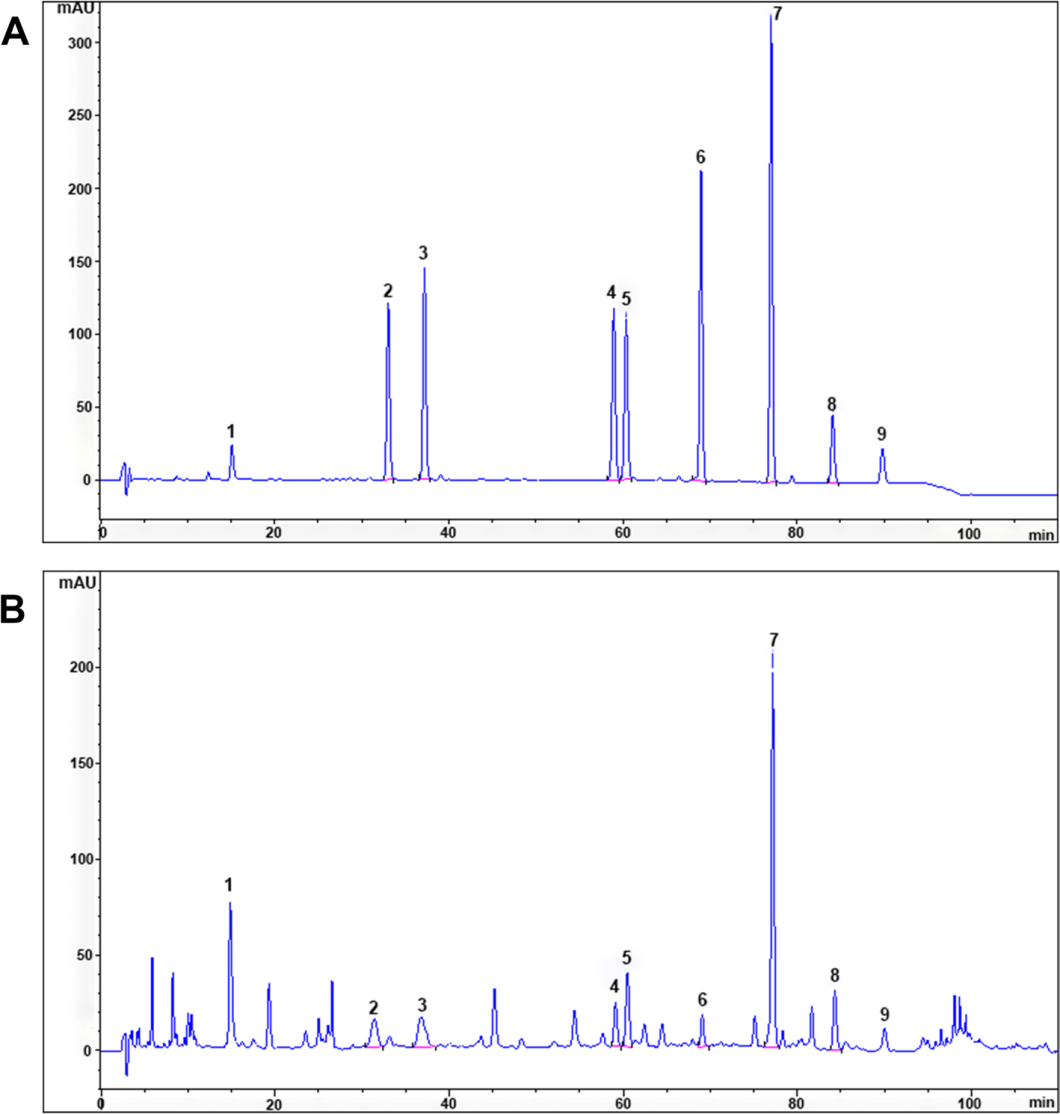
HPLC chromatogram of chloroform part of SHF. **A** The standard control. **B** The chloroform extract of SHF. 1. Dracorhodin perchlorate 2. Aloe-emodin 3. Rhein 4. Imperatorin 5. Emodin 6. Isoimperatorin 7. Chrysophanol 8. Physcion 9. Tanshinone IIA

Under the conditions of the “equipment and chromatographic conditions” experiment, the chromatogram was obtained by injecting the reference solution (Fig. 7). System suitability was determined by injecting a sample of the chloroform extract of SHF, including theoretical plates, and the resolution and tailing factor were calculated (Table 2). The calibration curves of 9 analytes were fitted with coefficients of determination greater than 0.999. The linear ranges were set as 12.90 to 258.00 μg/mL for dracorhodin perchlorate, 9.29 to 185.71 µg/mL for aloe emodin, 11.30 to 226.00 µg/mL for rhein, 6.55 to 131.00 µg/mL for imperatorin, 15.32 to 306.43 µg/mL for emodin, 4.42 to 221.00 µg/mL for isoimperatorin, 20.70 to 414.00 µg/mL for chrysophanol, 7.96 to 159.29 µg/mL for physcion, and 8.14 to 162.86 µg/mL for tanshinone IIA, according to the approximate concentrations of the sample. The relative standard deviations (RSD) of the precision, stability and repeatability tests were all less than 5%. The accuracy of the system was observed by recovery. The average of 9 analytes of the chloroform extract of SHF recoveries (*n* = 6) were dracorhodin perchlorate: 103.62% (RSD = 0.62), emodin: 101.87% (RSD = 2.42), rhein: 102.09% (RSD = 1.64), imperatorin: 102.55% (RSD = 1.72), emodin: 103.01% (RSD = 1.36), isoimperatorin: 102.80% (RSD = 2.11), chrysophanol: 102.06% (RSD = 2.16), physcion: 95.84% (RSD = 0.62), and tanshinone IIA: 99.33% (RSD = 2.67). The limits of detection (LODs) and limits of quantification (LOQs) were determined by using signal-to-noise ratios of 3:1 and 10:1. LOD and LOQ results of 9 analytes (Table 2). Three samples of the same batch of original medicinal materials were prepared by the “Preparation of standard and sample solutions of chloroform part for HPLC” method, and contents of 9 analytes (Table 2) were calculated by the regression equation.

**Table 2:** System suitability, LOD, LOQ and the concentration for 9 analytes

Analytes	Theoretical plate	Resolution	Tailing factor	LOD (µg/mL)	LOQ (µg/mL)	sample 1 (mg/g)	sample 2 (mg/g)	sample 3 (mg/g)	Mean (*n* = 3)	RSD (%)
Dracorhodin perchlorate	9233	1.74	1.416	0.37	1.29	3.42	3.54	3.41	3.46	2.18
Aloe-emodin	44,145	1.6	1.033	0.25	0.93	0.81	0.85	0.82	0.82	2.85
Rhein	50,103	3.35	1.082	0.27	1.13	1.04	1.07	1.03	1.05	1.74
Imperatorin	103,352	2	1.028	0.44	1.64	0.89	0.9	0.86	0.88	2.17
Emodin	98,885	1.48	1.045	0.69	1.39	1.95	2.03	1.96	1.98	2.12
Isoimperatorin	187,272	2.19	1.033	0.13	0.44	0.58	0.58	0.58	0.58	0.41
Chrysophanol	271,269	2.2	1.041	0.22	0.73	4.45	4.65	4.47	4.52	2.48
Physcion	270,813	1.49	1.069	0.36	1.32	1.24	1.29	1.25	1.26	2.27
Tanshinone IIA	225,496	4.05	1.046	0.14	0.45	0.57	0.6	0.57	0.58	2.87

## Discussion

The pathogenesis of diabetic ulcers remains unclear, and existing therapies include anti-infection, hyperbaric oxygen therapy, and surgery [15]. Wound healing is a complex process that includes fibroblast proliferation, angiogenesis and granulation tissue formation. Reducing the secretion of inflammatory factors, enriching collagen and promoting epidermal cells play an important role in different stages of diabetic ulcers [16–20]. External treatment with TCM has advantages in curing cutaneous ulcers and has been widely applied in China. Herbs regulate the immune [21] and skin microenvironments [22]. TCM has gradually been used to treat chronic ulcers and has achieved good effects [22–24].

SHF has been used to treat diabetic ulcers for over 30 years. Clinical studies have indicated that SHF can significantly improve diabetic ulcer wound healing by removing necrotic tissues and stimulating granulation tissues [25]. In our previous study, it was confirmed that SHF could reduce activin/follistatin protein levels, thus accelerating re-epithelialization during wound healing [11]. In addition, SHF could also reduce local inflammation by downregulating the protein expression of TNF-α, IL-1β, and IL-6 [9]. However, the effect of the exact active polar parts of this formula remains unclear. Few studies have focused on the active polar parts of SHF in treating diabetic wound healing. Due to the complicated pharmaceutical composition of SHF, finding the effective polar parts of SHF that treat diabetic ulcers is difficult. In this research, we further explored and compared different extractions of SHF and thus determined the potential active polar parts of this formula that treat STZ-induced diabetic ulcer rats.

The activity of active parts in SHF is closely related to basement membrane reconstruction and epithelial regeneration. At present, skin inflammation, epidermal proliferation, scar formation and tissue remodeling are thoroughly understood [26–28]. Astragaloside IV from *Astragalus membranceus* (Fisch.) Bge can promote wound repair in diabetic mice. The mechanism is mainly related to enhancing collagen deposition and extracellular matrix (ECM)-related gene expression, promoting angiogenesis and improving the expression of vascular endothelial growth factors [29]. Deoxyshikonin from *Angelica dahurica* enhances the migration of vascular endothelial cells, stimulates the phosphorylation of p38 and extracellular signal-regulated kinase, and thus accelerates wound healing [30, 31]. The chloroform extract of SHF was confirmed to be effective in treating diabetic ulcers. There were nine main components that synergistically improved wound healing in the chloroform extract. Among these components, dracorhodin and rhein were characteristic. Dracorhodin could promote the proliferation of fibroblasts [32] and keratinocytes [33] during wound healing. In addition, dracorhodin can inhibit the secretion of IL-1α and TNF-α, alleviate inflammation, stimulate the expression of vascular endothelial growth factors and TGF, and promote fibroblast proliferation and collagen deposition [34, 35]. Rhein could reduce inflammation, expediting angiogenesis, and promoting wound healing [36]. Therefore, further research on the main components in SHF might help to explore the most effective extract in the formula during diabetic wound healing.

In this study, STZ was injected into rats to establish a diabetic model. High-fat and high-sugar diets were fed to ensure the stability of the animal model. The positive control group used Bei Fuxin (rb-bFGF), which can promote vascular regeneration, improve local blood circulation, and accelerate wound healing and is used to treat burn wounds, chronic wounds and fresh wounds [37]. The measurement of wound area is the main parameter used to evaluate ulcer repair. Based on the results, no significant difference was observed between the treatment group and negative control group in the initial stage of wound treatment. During the inflammation stage, large amounts of inflammatory factors were discovered within the wound, which was not conducive to wound healing. However, from Day 9 of treatment, the effect of the positive group and chloroform group was significantly better than that of the negative control group. During this stage, abundant granulation regeneration could be found within the wound. The wound showed a faster closure rate. It has been reported [38] that the indices are width of wound healing and thickness of epidermis repair, which are used to judge the degree of wound healing. Complete repair of the epidermis, close connection of granulation tissues and vigorous angiogenesis are the primary conditions for wound healing. The results indicated that the wound healing degree of each group showed differences from the 9th day. Histology and HE staining showed that the wound healing degree of the positive control group and chloroform group was better than that of the negative control group, indicating that the positive control group and chloroform could promote wound healing. No significant difference was observed between the two groups. Therefore, chloroform extract could improve wound healing by accelerating granulation regeneration.

PCNA is an indication of the degree of cell proliferation and could help epidermal regeneration and wound repair. PCNA staining results showed that PCNA-positive cells were distributed in wound tissues of the negative control group, positive control group, chloroform group and *n*-butanol group. However, the expression of PCNA-positive cells in the positive drug group and chloroform group was higher than that in the negative control group. VEGF can enhance vascular permeability, regulate endothelial cell growth and promote cell migration [39, 40]. VEGF staining results showed that the VEGF-positive cells were accompanied by regularly arranged vascular endothelial cells in the chloroform group, which was more than that in the negative control group. Therefore, high expression of VEGF promoted granulation tissue formation and new angiogenesis. In cases of hypoxia, hypoxia-inducible factor-1α (HIF-1α) is known to be involved in mediating protein expression [41]. Additionally, keratinocytes are important cell types during wound repair [42]. It was reported that HIF-1α could regulate VEGF expression in human keratinocytes treated with chloroform [43]. Thus, we speculated that the chloroform extract of SHF might promote the generation of VEGF although mediating HIF-1α; however, more in-depth molecular studies are needed for confirmation.

Our HPLC results indicated that dracorhodin, aloe-emodin, rhein, imperatorin, emodin, isoimperatorin, chrysophanol, physcion, and tanshinone IIA were the main components of the chloroform extract from SHF. It was reported that dracorhodin could accelerate wound healing by facilitating the expression of VEGF and supporting collagen deposition [44]. Aloe-emodin was found to promote wound healing by regulating exosome release [45]. Rhein improved wound healing by decreasing inflammation and stimulating collagen deposition [46]. Similarly, imperatorin was confirmed to improve wound healing by increasing the secretion of VEGF, EGF and TGF-β1, thereby facilitating re-epithelization [47]. Tanshinone IIA was discovered to activate the PI3K/Akt/eNOS pathway, thus ameliorating wound healing [48]. As the abovementioned monomers were the main components of the chloroform extract from SHF and many of these monomers exhibited a capacity to promote wound healing, we therefore speculated that the chloroform extract might accelerate wound repair.

## Conclusion

In this study, the effective active parts of SHF were concentrated in chloroform by exploring the pharmacodynamic material basis of TCM. The nine main chemical components in the chloroform extract were determined by HPLC. By investigating chromatographic conditions, such as column, mobile phase, and detection wavelength, a method of evaluating the main chemical constituents in chloroform extract of SHF was established to provide a scientific basis to comprehensively and accurately evaluate the quality of medicinal materials. It was discovered that the chloroform extract of SHF could stimulate granulation tissues and improve the generation of PCNA and VEGF compared with petroleum ether, ethylacetate and n-butanol extracts of SHF. The chloroform extract was clarified to be the effective polar part. However, the molecular mechanisms of SHF in treating diabetic wounds remain unclear. The monomer compound of SHF ethanol extract-derived fractions on wound healing and potential specific markers for the recovery and outcome of diabetic wounds require more convincing evidence and research.

## Data Availability

The datasets supporting the conclusions of this article are included within the article.

## Abbreviations

SHF: Shengji Huayu Formula
STZ: Streptozotocin
HPLC: High performance liquid chromatography
VEGF: Vascular endothelial growth factor
IDF: The International Diabetes Federation
rb-bFGF: Recombinant bovine basic fibroblast growth factor
PCNA: Proliferating cell nuclear antigen
LSD: Least significant difference
ECM: Extracellular matrix
